# Chemical Composition and Antioxidant Properties of Essential Oils from Peppermint, Native Spearmint and Scotch Spearmint

**DOI:** 10.3390/molecules24152825

**Published:** 2019-08-02

**Authors:** Zhaohai Wu, Bie Tan, Yanhong Liu, James Dunn, Patricia Martorell Guerola, Marta Tortajada, Zhijun Cao, Peng Ji

**Affiliations:** 1 Department of Animal Science, University of California, Davis, CA 95616, USA; 2 State Key Laboratory of Animal Nutrition, College of Animal Science and Technology, China Agricultural University, Beijing 100193, China; 3 Key Laboratory of Agro-ecological Processes in Subtropical Region, Institute of Subtropical Agriculture, Chinese Academy of Sciences, Changsha 410125, China; 4 Applied Nutrition, ADM Animal Nutrition, Quincy, IL 62305, USA; 5 Cell Biology Laboratory, ADM Biopolis, 46980 Valencia, Spain; 6 Department of Nutrition, University of California, Davis, CA 95616, USA

**Keywords:** antioxidant activity, essential oil, spearmint, peppermint

## Abstract

Natural antioxidants have drawn growing interest for use in animal feed and the food industry. In the current study, essential oils (EOs) obtained from hydrodistillation of three *mentha* species, including *Mentha* *piperita* (peppermint), *Mentha spicata* (native spearmint) and *Mentha* *gracilis* (Scotch spearmint), harvested in the Midwest region in the United States, were analyzed for their chemical composition using gas chromatography-mass spectrometry, and their antioxidant properties were assessed through chemical assays, in vitro cell culture modeling and in *Caenorhabditis elegans* (*C. elegans*). The activity of ferric iron reduction and free-radical scavenging capacity were assessed through chemical-based assays, including the reducing power assay, 2,2-diphenyl-1-picrylhydrazyl (DPPH) radical scavenging assay, and Trolox equivalent antioxidant capacity assay (TEAC). Subsequently, the capacity of EOs to mitigate lipid peroxidation was analyzed at various doses using fresh liver homogenates from pigs. A porcine jejunum epithelial cell line (IPEC-J2) was employed as in vitro model to study the cellular antioxidant activity of the mint EOs. Finally, the effectiveness of mint EOs to alleviate acute systemic oxidative damage were evaluated in vivo using *C. elegans*. Data were analyzed by the MIXED procedure of SAS. Contrast statement was performed to assess linear or quadratic effects of mint EOs given at various doses. All three EOs are mostly composed of monoterpenes and their derivatives (76–90%), but differed in the major compounds, which are menthol and menthone (50%) in peppermint EO and carvone (70%) in spearmint EOs. Three mint EOs demonstrated prominent radical scavenging and Fe^3+^ reducing activity in chemical-based assays. In comparison with native and Scotch spearmint EOs, peppermint EO had the lowest (*p* < 0.05) half maximal effective concentration (EC50) in DPPH and TEAC assays and higher efficacy in the reducing power assay. All three EOs exhibited equivalent activity in mitigation of chemical-induced lipid peroxidation in liver tissues in a dose-dependent manner (linear, *p* < 0.001). The maximal cellular antioxidant activity (CAA) was observed at 5 µg/mL for peppermint, and 100 µg/mL for native and Scotch spearmint EOs. The addition of 25 µg/mL of both spearmint EOs increased (*p* < 0.05) cellular concentrations of glutathione in H_2_O_2_-treated IPEC-J2 cells, suggesting enhanced endogenous antioxidant defense. Supplementation of 100 µg/mL of peppermint or Scotch spearmint EO significantly increased (*p* < 0.05) the survival rate of *C. elegans* in response to H_2_O_2_-induced oxidative stress. The protective effect is comparable to that of supplementation of 10 µg/mL of ascorbic acid. However native spearmint EO failed to reduce the death rate within the same supplementation dose (10–200 μg/mL).

## 1. Introduction

Intracellular reactive oxygen species (ROS) are normally present at low concentrations as byproducts of mitochondrial respiration and have beneficial effects on cellular processes. However, excessive ROS exposure causes oxidative damage to the lipid membrane, protein and DNA, which may lead to cell death and tissue injury [1,2]. The epithelial layer of the gastrointestinal tract is susceptible to oxidative damage as it is frequently exposed to noxious environmental factors (e.g., oxidized dietary constituents and pathogens). A large number of resident immune cells aligned with the gut epithelium is another significant source of ROS. Moreover, the insults from environmental stress factors (i.e., heat-stress) also heighten the production of ROS in enterocytes and compromises barrier function of gut epithelium in animals and humans [3,4,5,6]. In the livestock industry, oxidative stress has been recognized as one of the major threats to animal welfare, productive performance and the quality of animal products [3,7,8]. Feed use antioxidants is not only an effective approach to prevent autoxidation of dietary constituents but also a promising strategy to mitigate enteric oxidative stress in animals. Due to the consumer resistance to synthetic antioxidants (i.e., butylated hydroxytoluene, butylated hydroxyanisole and ethoxyquin) [9,10,11], identification of natural antioxidants has drawn growing interest. 

Essential oils (EOs) are liquid mixtures of volatile compounds that are commonly collected through steam distillation of aromatic plants. Essential oils have been reported for their beneficial biological functions including antiviral, antimicrobial, anti-inflammatory as well as antioxidant effects [12,13,14,15]. In spite of tremendous chemical diversity, most essential oils contain compounds that belong to two structural families, namely terpenoids and phenylpropanoids [16]. Both families consist of a large number of phenolic compounds that vary in their antioxidant activities [17]. Environmental factors and maturity of the plants have been shown to significantly the composition of EOs, and thus alter their antioxidant capacities. Therefore, it is critical that chemical profiles are analyzed when the antioxidant activities of EO products are compared. In general, phytochemicals act as antioxidants through several modes of action that involve: (1) retarding the formation of and/or scavenging free radicals; (2) chelating redox-active metals; (3) enhancing endogenous anti-oxidative defense and (4) reducing autoxidation [18,19,20]. 

*Mentha* (mint) is a genus of an aromatic perennial herb belonging to *Lamiaceae* family [21]. The peppermints (*Mentha × piperita L*.) and two species of spearmints, ‘Scotch’ spearmint (*Mentha* × *gracilis Sole*) and ‘Native’ spearmint (*Mentha spicata L*.) are among the most important crops in EO production worldwide, and have been widely used as flavors in food, toothpaste, pharmaceuticals and cosmetics [22]. Owing to the abundant content of phenolic compounds [23,24], aqueous extracts and EO from mint plants are potential natural antioxidants [25,26]. For instance, peppermint EO was found to be an effective alternative short-term treatment of irritable bowel syndrome in humans, of which the effect is considered to be mediated through its antioxidant and anti-inflammatory activities [27]. However, many studies that have assessed the antioxidant activities of mint Eos have exclusively relied on chemical-based assays, whereas the effectiveness of mint EOs in prevention of oxidative stress at cellular level or in a living organism have not been clearly characterized. This study aimed to thoroughly evaluate antioxidant properties of EOs collected from peppermint, native spearmint and Scotch spearmint using 3-tiered analyses, namely chemical assays, in vitro cell culture modeling and in vivo animal modeling in *Caenorhabditis elegans*. The general antioxidant activities in terms of iron reduction and free radicals scavenging were analyzed through chemical-based assays. The capacity of mint EOs in mitigating lipid peroxidation was subsequently determined at various doses using freshly collected pig liver homogenates. Then, a porcine jejunal epithelial cell line was used as an in vitro model of intestinal epithelium to assess the cellular and intracellular antioxidant functions of mint EOs. Finally, nematode, *Caenorhabditis elegans*, was used to validate antioxidant activity of EOs in vivo.

## 2. Results and Discussion

### 2.1. Chemical Composition of Mint EOs and Total Phenolic Compounds

Substantial differences in chemical composition and abundance were observed between peppermint and the two spearmint EOs (Table 1). Peppermint EO consisted of high amounts of menthol (38.45%), menthone (21.8%), 1,8-cineole (5.62%), and neo-menthol (4.19%, Table 1), which altogether represented 70% of non-polar compounds identified. Our results are consistent with other studies, wherein menthol and menthone were the most abundant constituents in peppermint EO [28,29]. Both Native and Scotch spearmint EOs are rich in carvone, a monoterpene ketone that is responsible for the aroma and the beneficial effects of the spearmint EOs [30]. In the current study, carvone (70.36 and 70.91% in native and Scotch spearmint EO, respectively) was the predominant compound followed by limonene (6.96 and 13.96%). Buleandra et al. [31] also reported the same primary compounds in the EOs of peppermint and spearmint harvested in Romania, but the concentration of carvone in the spearmint EO was only 51.7%. Both peppermint and spearmint are temperate plants and require long days for deposition of EO constituents. Therefore, geographical site and leaf maturity at harvest considerably affect chemical composition of mint EOs and their bioactivities including antioxidant capacity [31,32,33]. A wide range of carvone content (49.6% to 76.6%) was reported for EOs of spearmint harvested from 26 sites in the North_west Himalayan region of India [33]. In essential oil, it is believed that some unsaturated terpenes (i.e., terpinenes), monocyclic terpenes (i.e., thymol) and monoterpenes (i.e., limonene, 1,8-cineole, and carvone) contribute to the antioxidant activity [34]. 

### 2.2. Chemical-Based Antioxidant Activity Assays

The antioxidant effects of phytochemicals generally act through scavenging free radicals, chelating divalent metals, donating hydrogen or electron, and facilitating decomposition of peroxyl radicals, thus they could retard the formation of free radicals, slow or inhibit autoxidation process (chain-breaking antioxidant), or expedite the termination of autoxidation. A myriad of studies have revealed the potent antioxidant effects of EOs extracted from aromatic plants [16]. 

**Figure 1:**
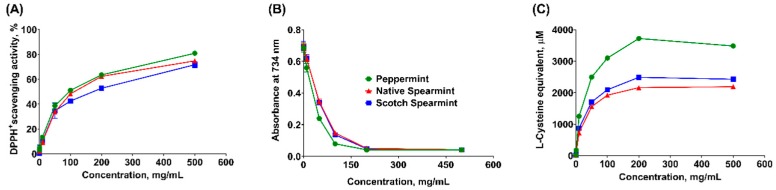
Dose response of mint essential oils using chemical-based antioxidant capacity assays. (**A**) 2,2-diphenyl-1-picrylhydrazyl (DPPH) radical scavenging activity assay, (**B**) Trolox equivalent antioxidant capacity (TEAC) assay, and (**C**) Reducing power assay. Data are least squares means of 6 observations per treatment.

In the current study, free-radical scavenging capacity of mint EOs was assessed through the 2,2-diphenyl-1-picrylhydrazyl (DPPH) radical scavenging assay. A dose-dependent increase of radical scavenging activity was observed in all mint EOs (Figure 1A) and the maximum response was reached at the highest concentration (500 mg/mL) of each mint EO. Peppermint EO had the lowest EC50 compared to both spearmint EOs (*p* < 0.05, Table 2), while the EC50 of Scotch EO was greater than that of native spearmint EO (*p* < 0.05). These results indicated the strongest radical scavenging activity of peppermint EO.

The 2,2′-azinobis [3-ethylbenzothiazoline-6-sulfonic acid]-diammonium salt (ABST) cation in Trolox equivalent antioxidant capacity (TEAC) assay has been widely used to determine antioxidant property with respect to the hydrogen-donating and chain breaking activities [35], which is expressed as Trolox equivalent in the current study. As shown in Figure 1B, the slopes of the curves were continuously decreased with the increase of concentration of mint EOs. Plateau was reached at 200 mg/mL for all three products (Figure 1B). In agreement with the DPPH assay, peppermint EO exhibited the lowest EC50 (*p* < 0.05), suggesting the strongest ABST cation scavenging activity (Table 2).

Free radical chain reactions can be terminated by a hydrogen or electron donor, and the precursors of peroxide can be removed by reductants [35]. Reducing power assay evaluates the capacity of electron donation of mint EOs by measuring the effectiveness in reducing ferric iron to its ferrous form [36]. With the increased concentration, all three mint EOs displayed increased reducing power (Figure 1C), which reached a plateau at 200 mg/mL (Figure 1C). Although the EC50 was not statistically different among three mint EOs, the maximum capacity of ferric iron reduction was the highest in the peppermint EO. 

As expected, all three mint EOs exhibited consistent anti-oxidative activity across different chemical-based tests. These observations were in agreement with studies in which the antioxidant activity of the mint EOs were observed and attributed to the major monoterpenoids including menthol, menthone, carvone and 1,8-cineole [37,38,39]. Other minor constituents in mint EOs that contain substances in the active methylene group, such as terpinolene, α- and γ-terpinene, were also reported for their strong antioxidant activity, which is comparable to α-tocopherol [17]. However, results from chemical-based assays should be interpreted with caution. Lack of standardized protocol in chemical assays results in inconsistent outcomes from different studies [40]. Furthermore, the concentration of mint EOs used in chemical assays may not be practical in biological systems. In particular, chemical assays can neither elucidate the bioactivities of different phytochemicals nor measure the indirect antioxidant activity (i.e., alter intracellular antioxidant enzymes) in a living organism. Therefore, we further evaluated antioxidant activities of mint EOs in fresh tissue homogenates, cell-based tests and in vivo model using *C. elegans*.

### 2.3. Lipid Peroxidation Assay

**Figure 2:**
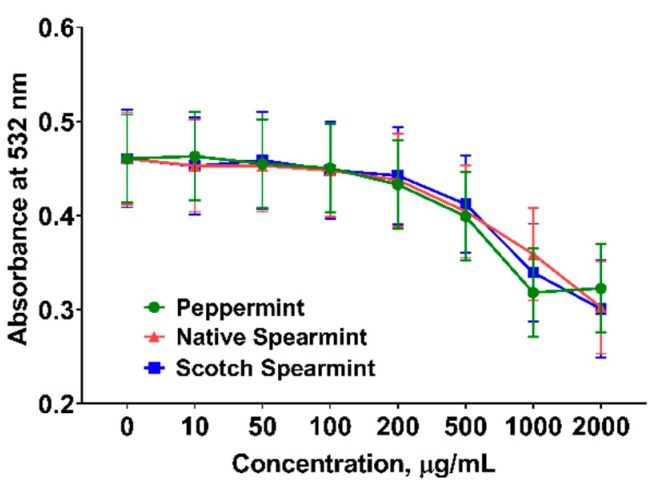
Inhibition of chemical-induced lipid peroxidation in pig liver homogenates by essential oils. Essential oils of peppermint, native spearmint, and Scotch spearmint dose dependently reduced (linear, *p* < 0.001) lipid peroxidation in liver. No differences were observed among 3 mint oils. Data are least squares means of 4 observations per treatment.

Lipid peroxidation is a process that oxidants (i.e., free radicals) attack lipids containing unsaturated fatty acids and generate lipid peroxyl radicals and hydroperoxides. The lipid layer(s) of the cellular membrane is critical in maintaining cell permeability and physiological homeostasis, but also vulnerable to lipid peroxidation which, if uncontrolled, may lead to cell death and tissue injury. The liver is the major metabolic organ that is particularly susceptible to lipid peroxidation damage. In the current experiment, we assessed the inhibitory effects of mint EOs on chemical-induced lipid peroxidation in fresh liver homogenates (Figure 2). All mint EOs similarly reduced (linear, *p* < 0.001) lipid peroxidation in a dose-dependent manner. The strongest inhibitory effect was observed at 1000 µg/mL for all mint oils. There was no further reduction of lipid peroxidation when mint oils were used at 2000 µg/mL. It has been reported that natural phenolic compounds (i.e., tocopherols) behave as pro-oxidants at high concentrations [41], which may explain the diminished inhibitory effect of mint oils on lipid peroxidation at 2000 µg/mL in the current study. 

### 2.4. Cellular-Based Antioxidant Activity Assay

**Figure 3:**
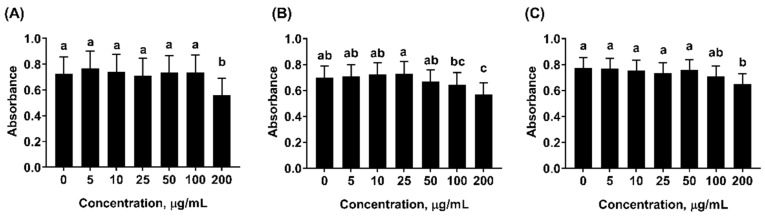
Effects of mint essential oils (EOs) on cell viability of porcine epithelial cells (IPEC-J2) in vitro. (**A**) Peppermint EO, (**B**) Native spearmint EO, and (**C**) Scotch spearmint EO. Data were expressed as least squares means of 6 observations. Values without common letter were significantly different (*p* < 0.05).

The intestinal epithelial layer is constantly exposed to oxidative challenges (e.g., oxidized feed and pathogens), and thus is susceptible to oxidative damage. In-feed use of antioxidants should also be effective in mitigation of epithelial oxidative stress. Hence, a porcine jejunal epithelial cell line (IPEC-J2) was used as an in vitro model to assess cellular antioxidant activity (CAA) of mint EOs. Prior to the CAA assay, the cytotoxicity of mint oils on IPEC-J2 cells was evaluated at various concentrations using of 3-(4,5-dimethylthiazol-2-yl)-2,5 diphenyltetrazolium bromide (MTT) assay. As shown in Figure 3, there was significant reduction in cell viability when the concentration of mint EOs reached 200 μg/mL. Essential oils of different *mentha* species had shown cytotoxic effects on cancer cells (e.g., gastric cancer SGC-7901, lung carcinoma SPC-A1, prostate cancer cells) as reported in other studies [32,42]. In addition, essential oil of *Mentha piperita* L. had shown moderate toxicity on larvae of *Artemia salina* (LC_50_ of 414.6 µg/mL) [43]. However, few studies have reported cytotoxic effects on gut epithelial cells. 

**Figure 4:**
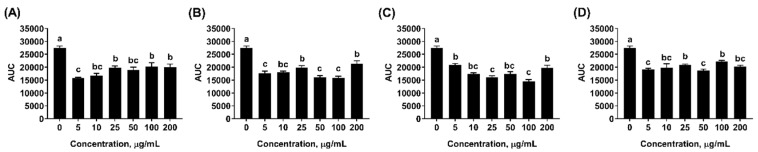
Cellular antioxidant activities of mint essential oils. Total area under curve (AUC) was computed using Trapezoid rule based on kinetic fluorescence data. The lower AUC indicated greater cellular anti-oxidative activity. (**A**) Peppermint EO, (**B**) Native spearmint EO, (**C**) Scotch spearmint EO, and (**D**) Trolox. Data were expressed as least squares means of 6 observations. Values without common letter were significantly different (*p* < 0.05).

Testing cellular antioxidant activity is biologically more meaningful than chemical-based antioxidant assays [44,45]. Dichlorofluorescin (DCF) is a probe that can be easily taken up by cultured cells. In the presence of intracellular peroxyl radicals, DCF will be oxidized to fluorescent dichlorofluorescein. The capacity of mint EOs to remove experimentally-induced intracellular peroxyl radicals and inhibit the formation of fluorescent DCF directly correlates with the antioxidant property of EOs [44]. In other words, the cumulative fluorescent signals quantified as area under curve (AUC) during the reaction period is negatively correlated with the antioxidant capacity of mint EOs. Supplementation of all three mint EOs in culture media significantly decreased the AUC (*p* < 0.05) compared to non-supplemented control samples, suggesting the protective effect of mint EOs against cellular oxidative damage (Figure 4). The cellular antioxidant effects of mint EOs was comparable to that of Trolox, a water-soluble analog of vitamin E. However, the maximum inhibition occurred at different concentration for each mint oil. Peppermint oil had maximal inhibitory effect when supplemented at 5 µg/mL, whereas the maximal inhibitory effect was not observed in spearmint and Scotch oil until the concentration increased to 100 µg/mL. The fact of compromised cellular antioxidant effects with higher concentration of EOs further supported that the pro-oxidant activity of mint EOs is concentration dependent. Unsaturated terpenes, such as linalool, γ-terpinene and α-pinene, are subject to autoxidation and generate reactive alkyl radical that bears pro-oxidant activity [16,17]. The higher contents of linalool and γ-terpinene in peppermint than the other two EOs might contribute to the greater pro-oxidant action of peppermint EO. Chen et al. [46] also reported that low doses of carvacrol (107 and 214 µM) reduced oxidative stress in Caco-2 cells, but high doses of carvacrol (460 µM) increased ROS level in those cells.

#### Intracellular Antioxidant Activity Assay

**Figure 5:**
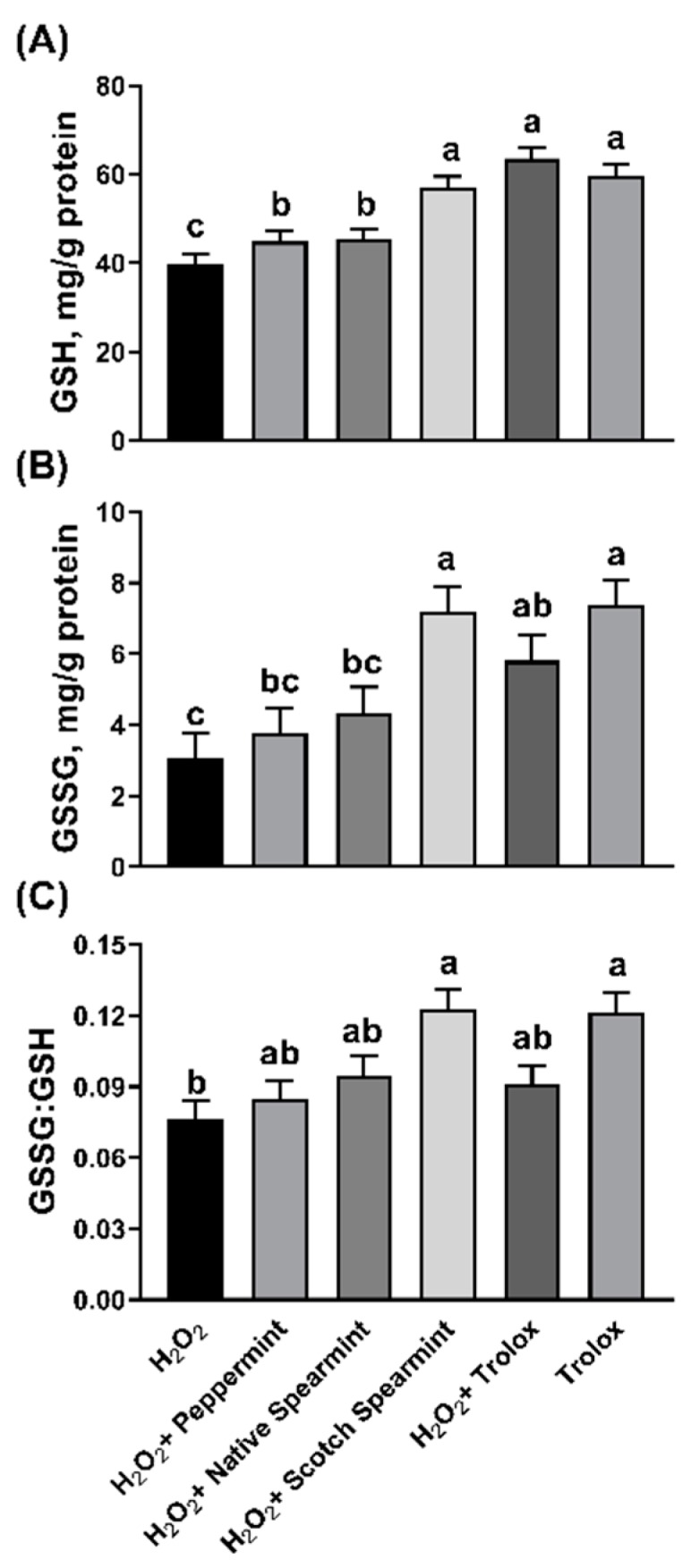
Effects of mint essential oils on the intracellular oxidation state of glutathione. IPEC-J2 cells were pre-treated with or without 25 µg/mL of mint oils or 1 µM of Trolox and incubated with 0.5 mM of H_2_O_2_. Concentration of clutathione (GSH, **A**), oxidized glutathione/glutathione disulphide (GSSG, **B**) and GSH to GSSG ratio (**C**) in cell lysates were analyzed. Data were expressed as least squares means of 4 observations. Values without common letter were significantly different (*p* < 0.05).

Glutathione (GSH) acts as an electron donor to directly scavenge free radicals or provides a substrate for glutathione peroxidase and glutathione *S*-transferase [47]. Glutathione (GSH) is oxidized to glutathione disulfide, an oxidoreduction process that quenches the intracellular free radicals associated with lipid hydroperoxide and hydrogen peroxide. The concentration of cellular GSH had significant bearing with total antioxidant capacity of cells. Aherne et al. [48] and O’Sullivan et al. [49] reported that sage and seaweed extracts which are rich in flavonoids increased the GSH content in Caco-2 cells. Based on the cell viability results and CAA results, an optimal dose of each mint oil (25 µg/mL) and Trolox (1 µM) was selected to conduct the intracellular antioxidant activity (IAA) assay. Hydrogen peroxide, a chemical inducer of cellular oxidative stress, significantly increased GSH concentration in IPEC-J2 cells than that of negative control (*p* < 0.05), highlighting the activation of protective response to neutralize H_2_O_2_-induced ROS. Supplementation of mint EOs and Trolox further increased (*p* < 0.05) cellular GSH, as well as glutathione disulfide (GSSG) concentration in H_2_O_2_-stimulated cells with the exception of peppermint EO (Figure 5). The effectiveness of both spearmint and Scotch EOs was comparable to that of Trolox. Similarly, Zou et al. [50] also observed that oregano mint EO elevated the expression of GSH in IPEC-J2 cells. The higher amount of GSSG in spearmint and Trolox treated cells might be resulted from increased conversion of GSH. However, neither mint EOs nor Trolox significantly changed the cellular GSSG:GSH ratio in H_2_O_2_-stimulated cells compared to positive controls (H_2_O_2_-stimulated cells). Unexpectedly, peppermint EO did not enhance GSH production, despite its superior antioxidant activity in chemical-based assays and overall effectiveness in mitigating cellular oxidative stress in vitro. Our finding is in contrast to the result of another study, wherein peppermint EO showed a prominent protective effect against carbon tetrachloride-caused hepatotoxicity in rats, as mediated through increasing the activity of superoxide dismutase (SOD) and GSH [51]. Analysis of antioxidant effectiveness of individual EO compounds revealed moderate antioxidant index of the main components of peppermint (menthol and 1,8-cineole) and the two spearmint EOs (carvon and limonene), but low antioxidant capacity of menthone [17].

### 2.5. In Vivo Antioxidant Analysis with Nematode Model

**Figure 6:**
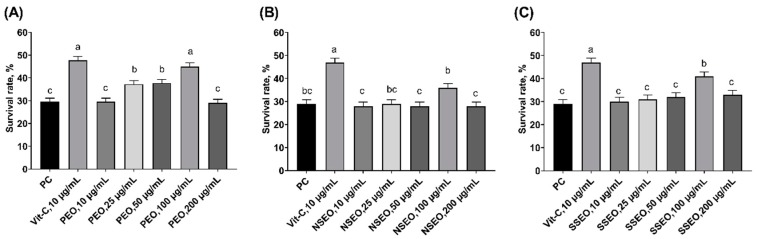
Effects of mint essential oils (EOs) on survival rate of C. elegans in response to H2O2-induced oxidative stress. The nematode C. elegans were grown in normal medium (PC), or medium containing 10 µg/mL of vitamin C, or different doses (10, 25, 50, 100, and 200 µg/mL) of peppermint EO (PEO, **A**), native spearmint EO (NSEO, **B**), and Scotch spearmint EO (SSEO, **C**). Data were expressed as mean ± SEM from duplicated trials. Ten replicate plates (10 worms/plate) were performed for each treatment within a trial. Values without common letter were significantly different (*p* < 0.05).

In the light of well-characterized endogenous antioxidant defense and short lifespan (~30 days), the nematode *C. elegans* is a widely-used animal model in the study of the antioxidant and antiaging effects of phytochemicals [52,53,54]. The presence of high concentrations of pro-oxidants (e.g., H_2_O_2_) and redox cycling chemicals (e.g., paraquat) have been shown to shorten the lifespan and increase mortality of *C. elegans* [55,56]. In the current assay, all worms were challenged with H_2_O_2_ to induce acute oxidative stress. Supplementation of 10 µg/mL vitamin C increased (*p* < 0.05) the survival rate of *C. elegans* by 18% compared with positive control (Figure 6). All mint EOs displayed pro-survival effects when supplemented at 100 µg/mL, but only peppermint EO enhanced survival rate to the extent that was comparable to 10 µg/mL of vitamin C. Moderate improvements of *C. elegans* survival (*p* < 0.05) were also observed when peppermint EO were supplemented 25 and 50 µg/mL. Although the mechanism behind the beneficial effects was not explored in the current study, peppermint EO has shown to revert the chemical-induced hepatotoxicity in rodent as mediated at least partially through its antioxidant properties [51]. Essential oil from *Juniperus communis L*. also displayed antioxidant and pro-survival effects as tested with *C. elegans*. Such beneficial effects were ascribed to its role in modulating insulin/IGF-1 signaling pathway [57]. It is unknown whether mint EOs function through the same mechanism. 

Our study employed multi-tests approach that holistically evaluated antioxidant effects of three mint Eos, including in vitro chemical- and cell-based assays and in vivo tests with *C. elegans* model. Collectively, all three mint-derived EOs showed significant antioxidant activity in chemical-based assays. The inhibitory effect against lipid peroxidation were comparable among three products. Peppermint EO, however, exhibited greater antioxidant capacity in DPPH, TEAC, and FRAP assays compared to EOs from native spearmint and Scotch spearmint. Furthermore, the maximal CAA response was observed at a much lower dose for peppermint EO (5 µg/mL) than for native or Scotch spearmint EOs (100 µg/mL). Results of IAA assay and in vivo *C. elegans* survival assays indicate that different cellular mechanisms may be involved in the antioxidant activity of peppermint, native spearmint, and Scotch spearmint EOs. Results of this experiment also suggested the potential use of all three mint EOs as alternative in-feed antioxidant in animal diets. The potential antibacterial effect [58] of mint EO may benefit feed preservation. However, proper processing techniques that could prevent loss of the volatile components of mint EOs is necessary because many of these compounds have antioxidant effects. More research should be conducted to verify the modes of action.

## 3. Materials and Methods

### 3.1. Preparation of Mint Essential Oil 

All three mint EOs were provided by ADM Animal Nutrition (Quincy, IL, USA). Mint EOs were produced through hydro-distillation of leaves from peppermint, native spearmint and Scotch spearmint that grew in Midwest region of the US and were harvested before the full flowering stage in summer 2016. The products from initial distillation were re-distilled to removes waxes and sulfur constituents.

### 3.2. Analyses of Chemical Composition

The total phenolic contents of the mint oils were determined using Folin-Ciocalteu reagent, as described by Singleton et al. [59]. Chemical composition (non-polar compounds) of EOs were analyzed by A. M. Todd Company (Kalamazoo, MI, USA) using GC-MS (Agilent 6890N inert gas chromatograph coupled with 5973 mass selective detector system) fitted with a fused silica SPB-1 non-polar capillary column (length 60 m × 0.25 mm ID, 0.25 μm film thickness; 24030-U, Sigma-Aldrich Co. LLC, St. Louis, MO, USA). Helium (average velocity 26 cm/s at constant flow) was used as a carrier gas. The oven temperature was held at 60 °C for 2 min and increased to 280 °C at a rate of 2 °C/min. Sample (1 μL) was injected at 250 °C with a split/split-less injector (50:1 split ratio). Multiple internal standards were used to normalize chromatographic data. Constituents of mint EOs were identified based on their mass spectra and retention data through library search including HPCH2205 and A.M. Todd databases and in comparison with mass spectra of authentic compounds. 

### 3.3. Chemical-Based Antioxidant Assays

Three chemical-based antioxidant assays were performed to evaluate antioxidant activity and free radical scavenging capacity, including DPPH radical scavenging capacity assay, Trolox equivalent antioxidant capacity (TEAC) assay, and reducing power assay. Each mint EO was tested at 0, 1, 10, 50, 100, 200, 500 mg/mL. Test samples were prepared by diluting the stocking solution with methanol (*w*/*v*) with the exception that 80% ethanol was used to prepare test samples for TEAC assay. All assays were repeated five times (replicates).

#### 3.3.1. DPPH Radical Scavenging Capacity Assay

The scavenging capacity of mint EOs against 2,2-diphenyl-1-picrylhydrazyl (DPPH, Sigma, St. Louis, MO, USA) radical was determined based on the method described by Zhou et al. [60]. Briefly, test sample was mixed with DPPH solution (25 µg/mL in methanol) at a ratio of 1:39 (*v*/*v*). Blank sample was prepared by mixing methanol with DPPH solution at the same ratio. The mixture was shaken to mix well and incubated at room temperature for 30 min. At the end of incubation, the absorbance at 517 nm was read immediately using a Synergy HTX Multi-Mode Microplate Reader (BioTek, Winooski, VT). The scavenging capacity of each mint EO was calculated based on the equation: DPPH^+^ scavenging capacity (%) = [(A_blank_ − A_test_)/A_blank_] × 100, where A_blank_ was the absorbance of blank sample, and A_test_ was the absorbance of test sample. Half maximal effective concentrations (EC50, mg/mL) of each mint EO were calculated accordingly. A lower EC50 indicates a higher radical scavenging capacity. 

#### 3.3.2. Trolox Equivalent Antioxidant Capacity Assay

The assay was performed based on the procedures described by Re et al. [61]. Briefly, potassium persulfate (2.45 mM) was mixed in 7 mM 2,2′-azinobis [3-ethylbenzothiazoline-6-sulfonic acid]-diammonium salt (ABST; Thermo Fisher, Petaluma, CA, USA) aqueous solution in the dark for 16 h to generate monocationic radical (ABST**^+^**) solution. The solution was diluted with 80% ethanol to adjust the absorbance to 0.7 (±0.05) at 734 nm. The test sample was mixed with ABTS^+^ solution at a ratio of 1:39 (*v*/*v*). The reactive mixture was allowed to stand at room temperature for approximately 5 min. The absorbance was subsequently measured at 734 nm. Trolox that prepared at different concentrations (0, 0.1, 0.5, 1, 5, 10, and 50 mM) were tested to generate standard curve. However, the optical density (OD) results of standard samples of Trolox fitted into a nonlinear curve, wherein the standard samples with concentration above 1 mM maximized the response. Therefore, it is inaccurate to calculate Trolox equivalent of the test samples that yielded OD of maximal response. The EC50 (mg/mL) of each mint EO were calculated based on OD values rather than calculated Trolox equivalent values. 

#### 3.3.3. Reducing Power Assay

The ferric iron reducing capacity of mint EOs was determined following the procedures by Oyaizu [62] and Oboh and Ademosun [63] with minor modifications. Briefly, equal volume of test sample, 2 M phosphate-buffered saline solution (PBS, pH 6.6) and 1% potassium ferricyanide (Sigma, St. Louis, MO) were mixed thoroughly. The solution was incubated at 50 °C for 20 min, and then was added with 10% trichloroacetic acid (Sigma, St. Louis, MO, USA) at the ratio of 1:3 (*v*/*v*) followed by centrifugation at 3000× *g* for 10 min. The supernatant was collected and mixed with an equal volume of 0.1% ferric chloride. Absorbance at 700 nm was measured immediately. l-cysteine prepared at different concentrations (0, 1, 5, 10, 50, and 100 µM) were used as the standard samples. The ferric reducing capacity was calculated as l-cysteine equivalent, and the EC50 was calculated for each mint EO. 

### 3.4. Lipid Peroxidation Assay

The capacity of mint EOs to mitigate lipid peroxidation were tested at 8 concentrations (0, 10, 50, 100, 200, 500, 1000, and 2000 µg/mL) following procedure by Oboh et al. [64] with minor modification. Fresh liver samples collected from 4 donor pigs were rapidly processed for lipid peroxidation assays. Liver tissue was firstly homogenized in ice-cold PBS (1:10 *w*/*v*) using Telfon tissue grinder. Tissue homogenate was centrifuged at 3000× *g* for 10 min to harvest lipid-containing supernatant. The lipid peroxidation was chemically induced using a mixture of FeSO_4_ and sodium nitroprusside (MP Biomedicals, Solon, OH). Reaction cocktail contains 100 µL of supernatants, 30 µL of 0.1 M Tris-HCl (pH = 7.4), 30 µL of 250 µM freshly prepared FeSO_4_, and 70 µM sodium nitroprusside and 100 µL test samples of mint EO or 1×PBS (negative control). The final volume was brought to 300 µL with water. The reaction cocktail was incubated at 37 °C for 1 h. Color reaction was developed by adding 300 µL 8.1% sodium dodecyl sulfate, 600 µL of acetic acid (pH 3.4), and 600 µL 0.8% thiobarbituric acid (Sigma, St. Louis, MO, USA). The mixture was further incubated at 100 °C for 1 h and recorded absorbance at 532 nm. Lipid peroxidation adduct of test samples were quantified by comparing the absorbance of the malondialdehyde (MDA) standard curve. 

### 3.5. Cellular-Based Antioxidant Activity Assay

#### 3.5.1. Cell Culture

Cellular antioxidant activity was tested using porcine intestinal epithelial cell line (IPEC-J2, DSMZ GmbH, Braunschweig, Germany). Cells were cultured at 37 °C with 5% CO_2_ in HyClone Dulbecco’s Modified Eagle Medium (DMEM with 4.5 g/L of glucose, Corning, Manassas, VA, USA) supplemented with 10% fetal bovine serum (Corning, Manassas, VA, USA), and 100 U/mL of penicillin and streptomycin. Cell culture medium was changed every other day and cells were passaged at 90% confluence. 

#### 3.5.2. Cell Viability Assay

Cytotoxicity of mint EOs were firstly assessed using MTT assay following a modified procedure from Liu et al. [15]. Briefly, IPEC-J2 cells were seeded in 96-well plates at a density of 1 × 10^4^ cells/well and incubated at 37 °C with 5% CO_2_ for attachment (~24 h). Cells were washed and treated with 100 µL culture media containing 0, 5, 10, 25, 50, 100, or 200 µg/mL of mint EO for 24 h. At the end of treatment, cells were washed, and 100 µL DMEM containing 10% (*v*/*v*) of 3-(4,5-dimethylthiazol-2-yl)-2,5 diphenyltetrazolium bromide (MTT, Thermo Fisher, Petaluma, CA) was added to each well. After 2 h of incubation, medium was replaced by 200 µL dimethyl sulfoxide (DMSO, Thermo Fisher, Petaluma, CA). The absorbance was measured at 570 nm with a reference wavelength at 630 nm. 

#### 3.5.3. Cellular Antioxidant Activity (CAA) assay

Cellular antioxidant activity of mint EOs were assessed at the concentration of 0, 5, 10, 25, 50, 100, and 200 μg/mL based on the procedure by Li et al. [65]. Briefly, IPEC-J2 cells were seeded (1 × 10^5^ cells/well) in a 96-well plates (black with transparent bottom) at 37 °C with 5% CO_2_ for 24 h. Cells were washed and then treated with 100 µL DMEM containing 50 µM dichloro-dihydro-fluorescein diacetate (DCFH-DA, Sigma, St. Louis, MO, USA) and mint EO at the proposed concentration for 30 min. After washing with PBS, cells were treated with 100 µL of 600 mM 2,2′-azobis(2-methylpropionamidine) dihydrochloride (ABAP) solution to form intracellular peroxyl radicals that oxidize DCFH to the fluorescent DCF. Dynamic fluorescence was measured using a plate reader for 13 cycles at 5 min intervals (λex = 485 and λem = 538) and subjected to blank subtraction (cells treated with DCFH-DA and PBS without oxidant). The area under the curve (AUC) of fluorescence versus time was integrated to calculate the CAA value of each sample as:

CAA (unit) = (1 − (ʃ SA/ʃ CA)) × 100(1)

where SA is the area of the test sample (cells treated with mint EO), and CA is the integrated area in the control sample (cells without EO treatment).

#### 3.5.4. Intracellular Antioxidant Activity (IAA) Assay

Based on the results of CAA assay, an optimal concentration of each mint EO (25 μg/mL) was selected to perform intracellular antioxidant assay. The IPEC-J2 cells were seeded in cell culture dishes (1 × 10^6^ cells/dish) in 1 mL DMEM at 37 °C with 5% CO_2_ for 24 h. After washing with 1 × PBS, cells were treated with either DMEM, 1 µM Trolox, or 25 μg/mL mint EO and incubated for another 18 h. Cells were further treated with 0.5 mM H_2_O_2_ for 1 h to induce cellular oxidative stress. Cells previously treated with DMEM were challenged with (positive control) or without H_2_O_2_ (negative control). Finally, cells were scraped off the culture dish, sonicated and deproteinated (subsamples were collected to normalize samples with total protein content before deproteination). Cell lysate was used to analyze the concentrations of glutathione disulfide (GSSG) and total glutathione (GSH) with a commercial Glutathione Assay Kit (Cayman Chemical, Ann Arbor, MI). The ratio of GSSG to GSH was calculated. 

### 3.6. In Vivo Antioxidant Activity in Caenorhabditis Elegans Model

#### 3.6.1. Cultures of Caenorhabditis Elegans (*C. elegans*)

The experiment was performed in the research center of Biopolis, S. L. (Valencia, Spain). Wild-type of strain N2 *Caenorhabditis elegans* was used as an in vivo model to evaluate antioxidant activity of the mint EOs. Nematodes were propagated on nematode growth medium (NGM; contains agar 17.5 g/L, sodium chloride 3.0 g/L, peptone 2.5 g/L, cholesterol 0.005 g/L) at 20 °C and fed *Escherichia coli* OP50 that was pre-seeded in the culture medium.

#### 3.6.2. Oxidative Stress Assays in *C. elegans*

In order to obtain synchronous cultures of nematodes, eggs were isolated from gravid adults and hatched overnight in NGM plates. Nematodes were cultured in plain NGM seeded with *E. coli* OP50 (positive control) or in the medium pre-mixed with vitamin C (10 µg/mL) or each mint EO at different concentrations (10, 25, 50, 100 and 200 µg/mL). Assays were performed as previously described by Martorell et al. [66]. Synchronized five days old adult nematodes were transferred to a basal medium containing 2 mM H_2_O_2_ and were remained in the medium for 5 h to induce acute oxidative stress. The survival of worms was observed under magnifying glass. Worms that were unresponsive to prodding were considered dead. Experiments were carried out in duplicates.

### 3.7. Statistical Analysis

Chemical-based assays, in vitro cellular antioxidant tests and in vivo nematode antioxidant tests were replicated for five, three and two times, respectively. Data were analyzed by means of one-way (comparison among conditions within each mint EO samples) or two-way (comparison among EO samples) ANOVA using PROC MIXED of SAS and adjusted for multiple comparison using Tukey’s test. Statistical significance and trend were claimed at *p* < 0.05 and 0.05 ≤ *p* < 0.10, respectively. 

## Figures and Tables

**Table 1:** Composition of non-polar constituents (peak area%) in mint essential oils (EO).

Compounds ^1^	Retention Time, min	Peppermint EO, %	Native Spearmint EO, %	Scotch Spearmint EO, %
Tricyclene	13.23	- ^2^	0.1	0.05
alpha-Pinene	14.03	0.56	-	-
1-Octen-3-ol	15.8	0.11	-	-
Sabinene	15.91	0.43	0.48	0.46
beta-Pinene	16.16	0.81	0.62	0.59
3-Octanol	16.82	0.26	1	2.68
beta-Myrcene	16.87	0.2	2.41	0.56
alpha-Terpinene	18.41	0.35	0.16	-
para-Cymene	18.6	0.32	0.22	-
1,8-Cineole	19.07	5.62	2.24	1.32
Limonene	19.31	1.58	6.6	12.99
cis-beta-Ocimene	19.53	0.34	0.2	-
gamma-Terpinene	20.91	0.56	0.36	0.05
trans-Sabinene Hydrate	21.15	0.86	1.1	0.14
alpha-Terpinolene	22.81	0.18	0.12	-
cis-Sabinene Hydrate	23.04	0.11	0.16	0.09
Linalool	23.21	0.37	0.09	0.07
Amyl Isovalerate	23.83	0.15	-	-
3-Octanol Acetate	24.84	0.05	0.32	0.13
trans-Pinocarveol	25.66	-	0.09	0.13
trans-Verbenol	26.02	-	-	0.11
Menthone	26.33	21.8	-	1.21
Isomenthone	26.91	3.75	-	0.16
Menthofuran	27.41	2.08	-	-
neo-Menthol	27.56	4.19	-	-
4-Terpineol	28.25	-	1.09	0.19
Menthol	28.59	38.45	0.12	-
iso-Menthol	28.78	0.71	-	-
cis-Dihydrocarvone	28.88	-	0.99	0.94
alpha-Terpineol	29	-	0.29	0.2
neoiso-Menthol/alpha-Terpineol	29.21	0.41	-	-
trans-Dihydrocarvone/Dihydrocarveol	29.29	-	0.44	0.11
neo-Dihydrocarveol	29.52	-	0.26	-
trans-Carveol	31.4	-	0.68	0.53
Pulegone	31.83	0.91	-	-
Carvone	32.59	-	70.36	70.91
Piperitone	32.86	0.65	0.26	0.31
Carvone Oxide (1st Isomer)	33.25	-	0.07	0.14
Carvone Oxide (2nd Isomer)	33.97	-	0.16	0.25
neo-Menthyl Acetate	34.96	0.22	-	-
Thymol	35.66	0.1	-	-
Menthyl Acetate	36.05	3.9	-	-
iso-Menthyl Acetate	37.03	0.19	-	-
Dihydrocarvyl Acetate	38.12	-	0.17	-
Eugenol	39.33	0.05	0.13	0.11
cis-Carvyl Acetate	40.24	-	0.7	0.12
cis-Jasmone	41.71	0.03	0.44	0.37
beta-Bourbonene	42.73	0.43	1.69	1.36
beta-Elemene	43.05	0.23	0.15	0.08
beta-Caryophyllene	44.8	2.87	1.1	0.88
beta-Copaene	45.37	0.07	0.25	0.18
trans-beta-Farnesene	46.88	0.49	0.97	0.44
Phenethyl Isovalerate	47.62	-	0.16	0.23
Germacrene D	48.47	3.24	1.01	0.5
Bicyclogermacrene	49.33	0.53	-	-
alpha-Muurolene	49.52	-	0.48	0.49
delta-Cadinene	50.78	0.16	0.05	0.06
Viridiflorol	54.51	0.8	0.45	-

^1^ Compound that accounts less than 0.1% of EO are not presented. ^2^ Non-detectable.

**Table 2:** Antioxidant activity of essential oils (EOs) of peppermint, native spearmint, and Scotch spearmint, measured by chemical-based antioxidant activities assays.

Assay ^1^		EC50, mg/mL	
	Peppermint EO	Native Spearmint EO	Scotch Spearmint EO
DPPH scavenging capacity	70.29 ± 4.59 ^c^	86.51 ± 5.45 ^b^	109.8 ± 6.70 ^a^
TEAC assay	29.51 ± 1.30 ^b^	45.74 ± 1.71 ^a^	44.38 ± 1.67 ^a^
Reducing power assay	22.7 ± 1.66	23.39 ± 2.66	22.91 ± 2.35

^a,b,c^ Within a row, means without a common superscript letter are different (*p* < 0.05). Data are least squares means of 6 observations per treatment. ^1^ TEAC = Trolox equivalent antioxidant capacity, FRAP = ferric reducing antioxidant power, EC50 = half maximal effective concentration, DPPH = 2,2-diphenyl-1-picrylhydrazyl.
